# A Case of Ertapenem-Induced Neurotoxicity in an Elderly Patient With Reduced Renal Function

**DOI:** 10.7759/cureus.83457

**Published:** 2025-05-04

**Authors:** Wyut Yi Htay, Wai Lin Aung, Wah Pwint Phyu

**Affiliations:** 1 Geriatrics, West Middlesex University Hospital, London, GBR; 2 General Medicine, West Middlesex University Hospital, London, GBR

**Keywords:** ertapenem, ertapenem-induced encephalopathy, geriatric medicine, neurotoxicity, reduced renal function

## Abstract

Ertapenem-induced neurotoxicity is a rare, often underreported side effect, primarily seen in elderly patients with impaired renal function. We report the case of an 87-year-old woman with a history of chronic kidney disease (CKD) and bilateral hydronephrosis, who presented with acute neurological symptoms, including left-sided weakness and slurred speech. Prior to this presentation, she had been treated for a left-sided nephrostomy site infection and left-sided perinephric collection with intravenous ertapenem at an ambulatory emergency care (AEC) center. An initial computed tomography (CT) scan of the brain showed no acute changes, but we treated her for a possible stroke while awaiting a magnetic resonance imaging (MRI) scan of the brain and continued intravenous ertapenem for the associated urological infection. However, the patient's condition continued to deteriorate, with the onset of further neurological symptoms, such as left-sided sensory deficits and constant myoclonic movements. An MRI scan of the brain showed no significant changes. The patient was diagnosed with ertapenem-induced neurotoxicity. We discontinued the antibiotic and switched to meropenem. Her neurological symptoms gradually improved and subsided within a week of stopping ertapenem.

This case highlights the importance of clinical suspicion of ertapenem-induced neurotoxicity in elderly and frail patients with reduced renal function who present with unexplained neurological symptoms. Prompt recognition of the condition facilitates accurate diagnosis and management. Treatment is simple and involves discontinuing the offending drug, leading to favorable outcomes.

## Introduction

Ertapenem, one of the beta-lactam antibiotics, is structurally a carbapenem, but its overall molecular structure has been modified to focus its antibacterial spectrum on community-acquired aerobic and anaerobic pathogens. This modification also increases its plasma half-life, permitting once-daily dosing for this parenteral antibiotic [1]. Aside from common side effects, ertapenem has been implicated in causing central nervous system (CNS) toxicity [2]. Neurotoxicity symptoms are considered rare, but cases with such symptoms are increasingly appearing within 3-7 days of starting the drug [3-5]. Patients with pre-existing renal impairment are more prone to neurotoxicity. Common symptoms include seizures, hallucinations, and altered mental status [6]. Cessation of the medication is the most important aspect of management, and symptoms usually resolve within 1-6 weeks [6]. In this case report, we discuss the clinical presentation, exclusion of potential causes, and management strategies in a patient presenting with ertapenem-induced neurotoxicity. This case report highlights the importance of recognizing a rare but reversible side effect of a commonly used antibiotic in clinical practice.

## Case presentation

**Figure 1:**
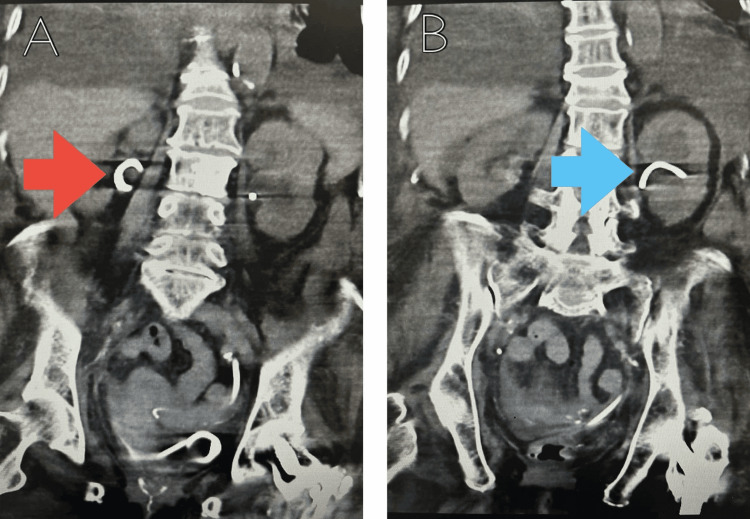
Coronal views of the CT scan of the urinary tract A: A coronal view of the CT scan of the urinary tract showing the right ureteric stent (red arrow). B: A coronal view of the CT scan of the urinary tract showing the left ureteric stent (blue arrow). CT: computed tomography

An 87-year-old woman weighing 42.6 kg, with a medical history of stage 3b chronic kidney disease (CKD), hypertension, atrial fibrillation, and heart failure, was initially hospitalized for bilateral hydronephrosis, complicated by acute kidney injury and an extended-spectrum beta-lactamase (ESBL) *Escherichia coli* urinary tract infection. During her admission, she underwent bilateral nephrostomies, which were later replaced by bilateral ureteric stents (Figure 1). She was discharged with instructions for urology follow-up and ambulatory emergency care (AEC) for monitoring of fluid status and renal function.

During AEC follow-ups, the previous left nephrostomy wound site became infected, and a renal ultrasound revealed a left perinephric collection. Therefore, an intravenous regimen of ertapenem 1 g once a day was initiated based on wound culture results and in consultation with microbiology. After four days of receiving antibiotic therapy, the patient presented to the emergency department with acute left-sided weakness and slurred speech. Examination revealed left-sided weakness, with motor strength of 3/5 in both the upper and lower limbs, accompanied by dysarthria. The remainder of the neurological examination was unremarkable, with no changes in mental status.

**Figure 2:**
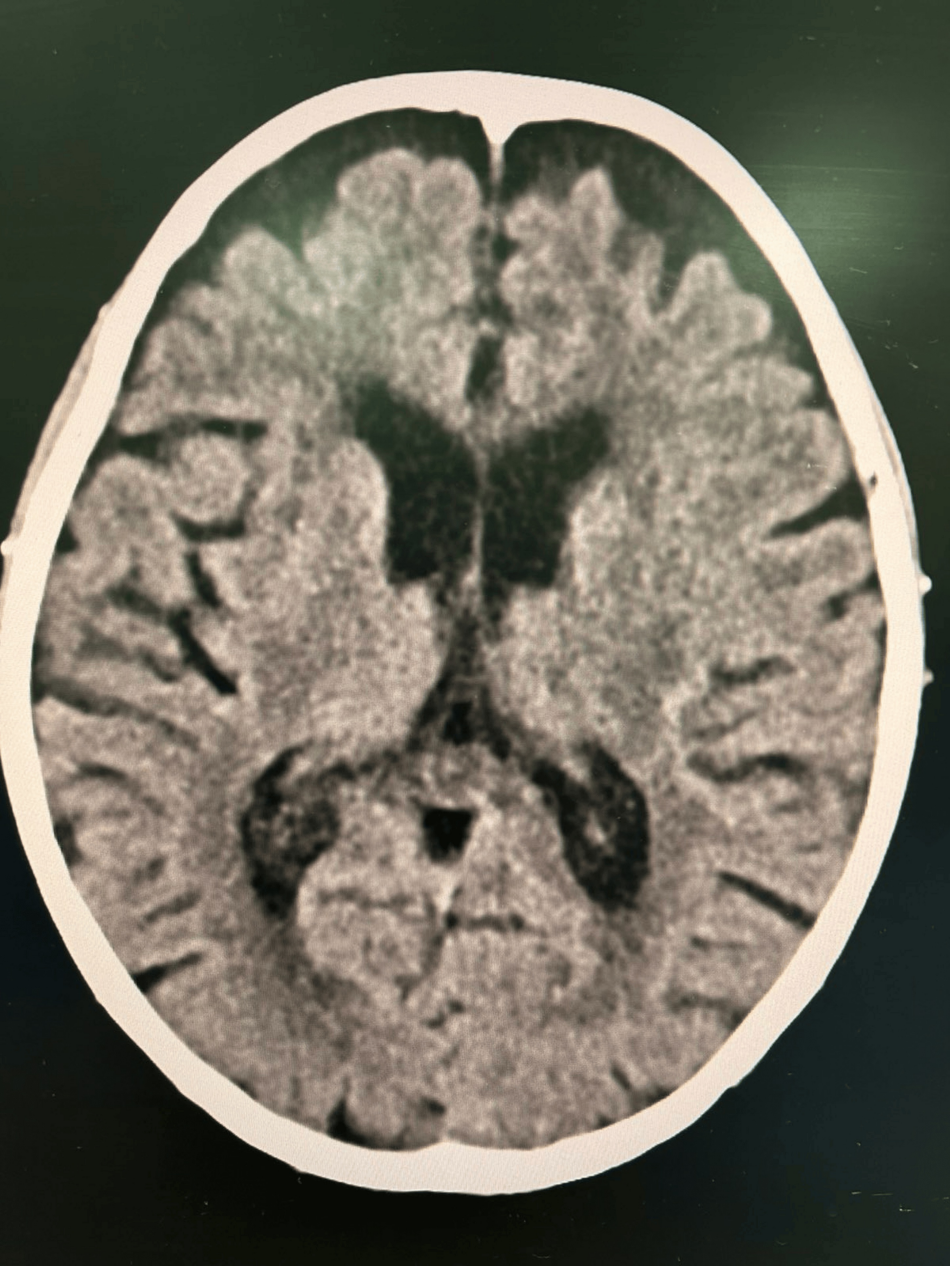
Axial view of the brain CT scan An unremarkable brain CT scan with evidence of age-related cerebral volume loss. CT: computed tomography

A computed tomography (CT) scan of the brain showed no acute changes (Figure 2), effectively excluding acute ischemic or hemorrhagic stroke, space-occupying lesions, and other acute structural abnormalities that could explain the patient's neurological symptoms. Routine blood tests indicated stable renal function, with an estimated glomerular filtration rate (eGFR) of 38 mL/minute/1.73 m². Other investigations, including electrolytes, glucose, vitamin B12, folate, and thyroid function tests, were within normal limits. Liver function tests were mildly elevated, and the serum albumin level was low (Table 1). Overall, the blood test results did not suggest metabolic, endocrine, or hepatic causes for the patient's presentation. Blood cultures were negative; however, urine cultures grew *Enterococcus*, and a wound swab from the previous left nephrostomy site revealed ESBL-producing *Escherichia coli*.

**Table 1:** Routine blood results of the patient on admission GFR CKD-EPI: Chronic Kidney Disease Epidemiology Collaboration (CKD-EPI) equation used to estimate GFR, GFR: glomerular filtration rate, g/L: grams per liter, mmol/L: millimoles per liter, umol/L: micromoles per liter, pmol/L: picomoles per liter, g/L: gram per liter, ug/L: micrograms per liter, ng/L: nanograms per liter

Parameters	Readings	Normal ranges
White cell count	5.8 × 10^9^/L	4.2-11.2 × 10^9^/L
Hemoglobin	103 g/L	114-150 g/L
Platelet count	159 × 10^9^/L	135-400 × 10^9^/L
Sodium	133 mmol/L	133-146 mmol/L
Potassium	4.6 mmol/L	3.5-5.3 mmol/L
Blood urea	8.5 mmol/L	2.5-7.8 mmol/L
Creatinine	111 umol/L	55-110 umol/L
Estimated GFR CKD-EPI	38 mL/minute/1.73 m^2^	>89 mL/minute/1.73 m^2^
Adjusted calcium level	2.32 mmol/L	2.2-2.6 mmol/L
Albumin level	24 g/L	35-50 g/L
Conjugated bilirubin	14 umol/L	0-5 umol/L
Alkaline phosphatase	146 unit/L	30-130 unit/L
Alanine aminotransferase	8 unit/L	0-34 unit/L
Aspartate aminotransferase	59 unit/L	0-40 unit/L
Thyroid-stimulating hormone	2.86 milliunit/L	0.30-4.20 milliunit/L
Free T4	16.3 pmol/L	9-23 pmol/L
Folate level	14.3 ug/L	>2.7 ug/L
Vitamin B12 level	425 ng/L	160-800 ng/L

**Figure 3:**
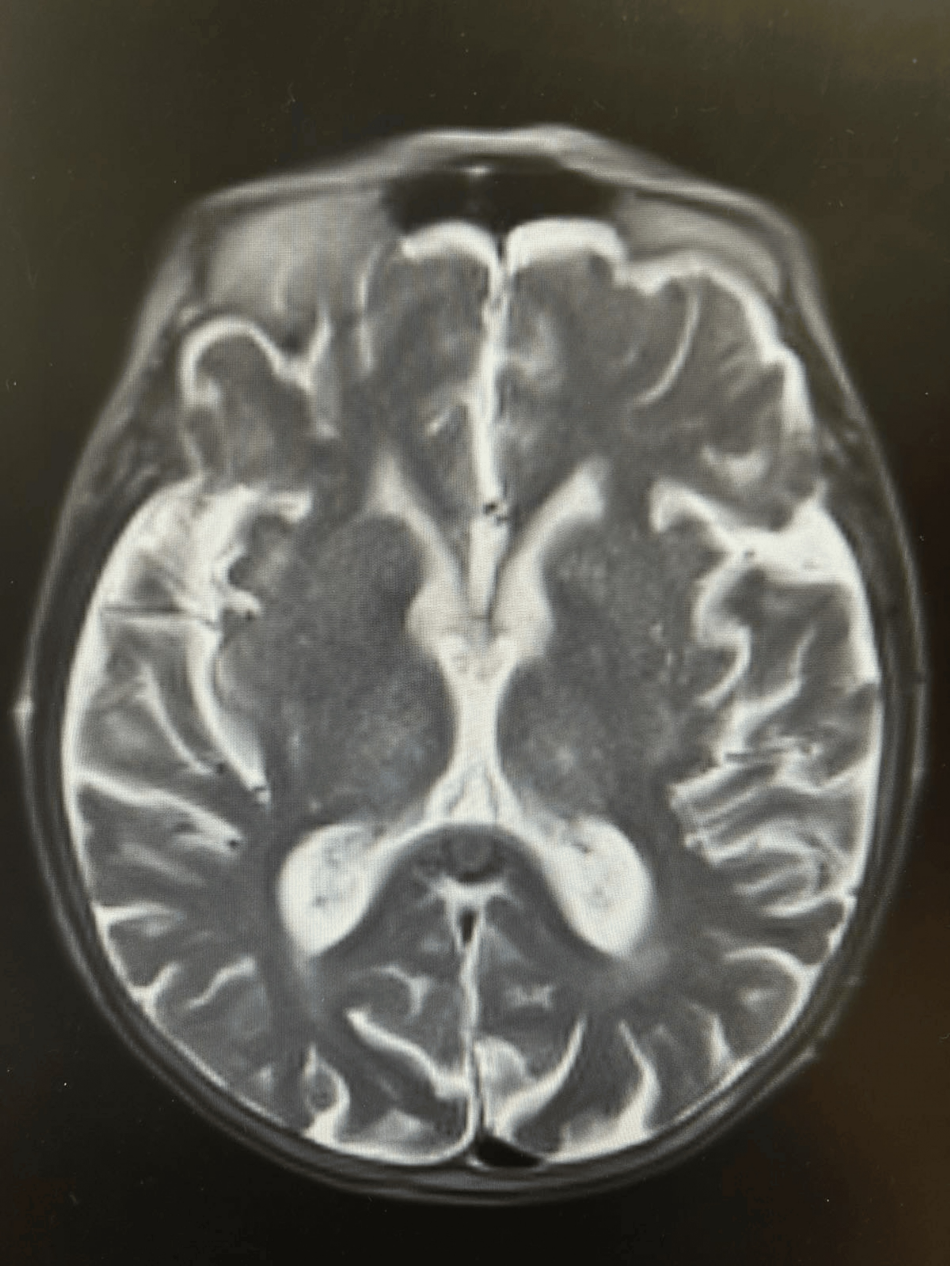
Axial view of the brain MRI scan An unremarkable brain MRI scan with evidence of age-related brain involutional changes. MRI: magnetic resonance imaging

In the following days, the patient continued to experience dizziness, reduced sensation in the left hand, asterixis, and persistent myoclonic movements in the left limbs. A magnetic resonance imaging (MRI) of the brain (Figure 3) revealed no significant abnormalities except age-related brain involutional changes.

We could not perform a lumbar puncture due to poor patient cooperation. However, no signs of fever, neck stiffness, or altered consciousness were present. Hypoxic encephalopathy was also unlikely, as there were no clinical or vital sign features suggestive of hypoxia, nor radiological findings supporting this diagnosis.

Given the unusual presentation and the absence of a clear diagnosis, the neurology and microbiology teams were consulted. After discussions, it was decided to discontinue ertapenem on the ninth day of therapy, as drug-related neurological effects were considered a possible cause of the symptoms. The patient had a Naranjo Adverse Drug Reaction (ADR) score of 5, which suggests a probable link between the drug and the reaction (Table 2). The patient was switched to meropenem 1 g twice a day, and levetiracetam 500 mg twice a day was started to manage the myoclonic movements. Within a week of stopping ertapenem, the patient's symptoms gradually subsided and resolved completely. An electroencephalogram (EEG) was performed 14 days after the discontinuation of ertapenem and showed no epileptiform discharges or myoclonus, by which time the patient's myoclonic jerking had resolved. Although an acute-phase EEG was not obtained due to clinical and logistical constraints, the timing of symptom resolution following drug cessation, along with the absence of alternative explanations, supported a diagnosis of ertapenem-induced neurotoxicity.

**Table 2:** Naranjo ADR Probability Scale Total score: 5 ADR: Adverse Drug Reaction

Question	Yes	No	Do not know	Score
Are there previous conclusive reports on this reaction?	1	0	0	1
Did the adverse event appear after the suspected drug was administered?	2	-1	0	2
Did the adverse event improve when the drug was discontinued or a specific antagonist was administered?	1	0	0	1
Did the adverse event reappear when the drug was readministered?	2	-1	0	0
Are there alternative causes that could, on their own, have caused the reaction?	-1	2	0	0
Did the reaction reappear when a placebo was given?	-1	1	0	0
Was the drug detected in blood or other fluids in concentrations known to be toxic?	1	0	0	0
Was the reaction more severe when the dose was increased or less severe when the dose was decreased?	1	0	0	1
Did the patient have a similar reaction to the same or similar drugs in any previous exposure?	1	0	0	0
Was the adverse event confirmed by any objective evidence?	1	0	0	0

## Discussion

Carbapenem antibiotics are crucial for treating both community- and hospital-acquired infections, particularly those caused by extended-spectrum beta-lactamase (ESBL)-producing bacteria, intra-abdominal infections, and skin and soft tissue infections. Among them, ertapenem has a longer half-life, enabling once-daily dosing, which is convenient for outpatient care in the UK. It also has higher plasma protein binding than other carbapenems such as imipenem and meropenem [1]. Common side effects of ertapenem include diarrhea, headache, nausea, skin reactions, thrombophlebitis, and vomiting, while rarer effects involve appetite loss, arrhythmias, pseudomembranous colitis, seizures, and confusion [2].

Neurotoxicity related to ertapenem is considered rare, but cases with neurological symptoms are increasing, typically appearing within 3-7 days of starting the drug [3-5] and resolving after discontinuation. Factors such as renal function, albumin level, dosage, age, body weight, and pre-existing neurological conditions can influence both the onset and duration of symptoms. Patients with renal impairment, especially those on dialysis, are more prone to ertapenem-induced neurotoxicity. In a study by Wang et al. (2023), 94% of patients who developed neurological symptoms had renal impairment, with the most common symptoms being seizures (42.4%), hallucinations (36.4%), and altered mental status (25.8%) [6].

Ertapenem-induced encephalopathy is thought to result from the inhibition of gamma-aminobutyric acid (GABA) receptor type A in the CNS [3], which is the major inhibitory neurotransmitter system. Animal studies suggest that about 2.4% of ertapenem crosses into the central nervous system, potentially causing neurological symptoms [7]. Hypoalbuminemia may also increase the risk of neurotoxicity, particularly seizures, compared to other carbapenems [7,8].

Based on the ertapenem product specifications, patients with creatinine clearance (CrCl) above 30 mL/minute/1.73 m² do not require dose adjustment. However, those with severe renal impairment, including those on dialysis, should receive a reduced dose of 500 mg daily. Ertapenem's half-life is prolonged in patients with end-stage renal disease (ESRD), leading to delayed drug clearance and increased penetration into the brain due to blood-brain barrier changes. In patients with ESRD, the half-life becomes 6.1-14.1 hours compared to the usual 4.5 hours in patients with normal renal function [9].

The Naranjo Scale is a good predictor of adverse drug reactions (ADRs); however, it is highly dependent on the evaluator [10]. Therefore, other relevant neurological differential diagnoses should be thoroughly investigated before concluding that ertapenem-induced neurotoxicity is the cause. The patient had a Naranjo Adverse Drug Reaction (ADR) score of 5, which suggests a "probable" link between the drug and the reaction. According to the Naranjo Scale, a score of 5-8 means "probable," 1-4 means "possible," and 0 or less means "doubtful." Therefore, in this case, the reaction is likely related to the drug.

A multidisciplinary team approach, including expert advice, should be sought whenever possible. The mainstay of treatment is cessation of the medication. Symptoms usually resolve within 1-6 weeks. Supportive care, such as sodium valproate, may be considered for severe cases, and hemodialysis can expedite drug clearance [6]. It is also important to assess renal function accurately, as overestimating renal function could lead to inappropriate dosing and increased risk of adverse effects. Variations in eGFR calculation methods across hospitals, particularly those that do not consider body weight, may result in dosing errors [11]. CKD-EPI equations can overestimate eGFR in patients less than 45 kg and underestimate eGFR in patients more than 80 kg; however, the gold standard is to calculate CrCl, as dose adjustments of medications are recommended based on CrCl.

Overall, the prognosis for ertapenem-induced neurotoxicity is good, with most patients experiencing full recovery after discontinuing the drug. The key management is early identification and stopping the medication, with close monitoring and supportive care when necessary.

Among the carbapenems, the risk of seizures from imipenem, meropenem, ertapenem, and doripenem compared with other antibiotics were 3.50 (95% confidence interval (CI): 2.23, 5.49), 1.04 (95% CI: 0.61, 1.77), 1.32 (95% CI: 0.22, 7.74), and 0.44 (95% CI: 0.13, 1.53), respectively [12]. The study mentioned above was focused on seizures, but the numbers were similar in other neurotoxicity studies as well.

## Conclusions

This case underscores the importance of clinical awareness regarding ertapenem-induced neurotoxicity, particularly in elderly individuals with reduced renal function who present with unexplained neurological symptoms. Although neurotoxicity secondary to ertapenem is rare, it should not be overlooked, given its potential to cause significant neurological impairment. The approach to diagnosis should first involve excluding other possible differential diagnoses and include a multidisciplinary team, comprising a neurologist, microbiologist, and pharmacist. Early recognition and discontinuation of the offending drug are essential for a favorable clinical outcome, as symptoms typically resolve after stopping the medication. We present this case to emphasize the need for heightened clinical awareness and careful monitoring, especially in vulnerable populations. Further research is needed to refine early detection strategies and evaluate the effectiveness of alternative treatments. Additionally, reporting of similar cases could help establish clearer clinical guidelines for managing such neurotoxic reactions in practice.
